# A new heavy lanthanide-dependent DNAzyme displaying strong metal cooperativity and unrescuable phosphorothioate effect

**DOI:** 10.1093/nar/gku1296

**Published:** 2014-12-08

**Authors:** Po-Jung Jimmy Huang, Mahsa Vazin, Żaneta Matuszek, Juewen Liu

**Affiliations:** Department of Chemistry, Waterloo Institute for Nanotechnology, University of Waterloo, Ontario, Canada N2L 3G1

## Abstract

*In vitro* selection of RNA-cleaving DNAzymes was performed using three heavy lanthanide ions (Ln^3+^): Ho^3+^, Er^3+^ and Tm^3+^. The resulting sequences were aligned together and about half of the library contained a new family of DNAzyme. These DNAzymes have a simple loop structure, and they are active only with the seven heavy Ln^3+^. Among the tested non-lanthanide ions, only Y^3+^ induced cleavage and even Pb^2+^ failed to cleave, suggesting a very high specificity. A representative DNAzyme, Tm7, has a sigmoidal metal binding curve with a Hill coefficient of 3, indicating that three metal ions are involved in the catalytic step. Its pH-rate profile has a slope of 1, suggesting a single deprotonation step is involved in the rate-limiting step. Tm7 has a cleavage rate of 1.6 min^−1^ at pH 7.8 with 10 μM Er^3+^. Phosphorothioate substitution at the cleavage junction completely inhibits the activity, which cannot be rescued by Cd^2+^ alone, or by a mixture of Er^3+^ and Cd^2+^, suggesting that two interacting metal ions are involved in direct bonding to both non-bridging oxygen atoms. A new model involving three lanthanide ions is proposed based on this study. A biosensor is engineered using Tm7 to detect Dy^3+^ down to 14 nM.

## INTRODUCTION

The interaction between lanthanide ions (Ln^3+^) and nucleic acids has been studied for a long time. At high lanthanide concentrations, both DNA and RNA can be cleaved (1). Therefore, Ln^3+^ and their complexes have been developed as artificial nucleases and chemical probes for nucleic acids (2–5). Tb^3+^ luminescence is significantly enhanced upon binding to DNA, and related biosensor and biochemical applications have been reported (6,7). Lanthanide ions are popular structural probes for ribozyme research as well, where they can sometimes replace the original metal ions and provide rich spectroscopic information (8). With strong hydrolytic properties, Ln^3+^ was exploited in RNA-cleaving DNAzymes. For example, Ln^3+^ can accelerate the leadzyme (a small Pb^2+^-dependent ribozyme) (9), but they inhibit the hammerhead ribozyme (8), and the 8–17 DNAzyme (2). Ln^3+^ moderately activates GR5 (a Pb^2+^-dependent DNAzyme) (10). The application of Ln^3+^ in other DNAzyme reactions was also reported (11,12).

We are interested in isolating new Ln^3+^-dependent DNAzymes. Using *in vitro* selection, we recently reported two new RNA-cleaving DNAzymes that are highly specific for Ln^3+^. The first one (named Ce13d) was selected in the presence of Ce^3+^/Ce^4+^ (13), and the second one (named Lu12) was selected in the presence of Lu^3+^ (14). Both are highly active with Ln^3+^, although Lu12 appears to be less active with the last few heavy ones. Using these two probes, we were able to discriminate only a few heavy Ln^3+^. Ideally, if more DNAzyme probes with distinct activity patterns across the Ln^3+^ series can be obtained, it may enable a sensor array to discriminate between each of the 15 lanthanide ions (15,16). In our effort to isolate more Ln^3+^-dependent DNAzymes, we herein performed three new selections using Ho^3+^, Er^3+^ and Tm^3+^ respectively. For all known elements, holmium has the largest magnetic strength and is used for making magnets. It is also used in nuclear reactors due to its ability to strongly absorb neutrons. Erbium is mainly used for making lasers and thulium is used in X-ray devices as a portable source.

From these selections, we identified a new class of DNAzymes with many interesting properties. For example, they work only with the seven heavy Ln^3+^. Different from all the previously reported examples (lanthanide or other metal dependent), this is the first RNA-cleaving DNAzyme showing metal cooperativity, indicating that multiple metal ions are involved for catalysis. Finally, phosphorothioate modification has resulted in complete inactivation of the DNAzyme, such that it cannot be rescued by adding thiophilic metals. With these observations, a trinuclear Ln^3+^ mechanism is proposed.

## MATERIALS AND METHODS

### Chemicals

The *in vitro* selection related DNA samples and the beacon DNA were purchased from Integrated DNA Technologies (IDT, Coralville, IA). See Supplementary Table S1 for their names, sequences and modifications. For characterization, the enzyme strands were from Eurofins (Huntsville, AL). The Ln^3+^ other metal salts were from Sigma–Aldrich at the highest possible purity. Tris(hydroxymethyl)aminomethane (Tris), 2-(*N*-morpholino)ethanesulfonic acid (MES), 2-[4-(2-hydroxyethyl)piperazin-1-yl]ethanesulfonic acid (HEPES), ethylenediaminetetraacetic acid (EDTA) disodium salt dihydrate, sodium chloride and ammonium acetate were from Mandel Scientific Inc. (Guelph, Ontario, Canada). SsoFast EvaGreen supermix was from Bio-Rad for real-time polymerase chain reaction (PCR). T4-DNA ligase, deoxynucleotide (dNTP) mix, Taq DNA polymerase with ThermoPol buffer and low molecular weight DNA ladder were from New England Biolabs.

### *In vitro* selection

The method of *in vitro* selection is similar to the one we reported previously (14). In brief, the initial library was obtained by ligating Lib-FAM-N_35_ and Lib-rA. For each subsequent round, the library was produced by PCR. For each cleavage step, the DNA library was incubated with fresh Ln^3+^ solutions. For all the selections, the metal incubation time was maintained at 60 min. For the first four rounds, 50 μM Ln^3+^ was used while for the last three rounds, 10 μM Ln^3+^ was used. After incubation, the solution was mixed with 8 M urea and purified by 10% dPAGE (denaturing polyacrylamide gel electrophoresis). The position corresponding to the cleaved product was excised from the gel, the DNA was extracted by crushing and soaking the gel and was further desalted with a Sep-Pak C18 column (Waters). After drying in an Eppendorf Vacufuge at 30°C overnight, the dried DNA was re-suspended in 70 μl of 5 mM HEPES buffer (pH 7.5). The round 6 libraries for all the three selections were cloned and sequenced.

### PCR

During the *in vitro* selection experiment, three PCR reactions were carried out for each round. After the cleavage step, real-time PCR was carried out to quantify the cleaved DNA extracted from the gel. Each 20 μl reaction mixture contained 1 μl purified DNA template, 400 nM primer (P1 and P2) and 10 μl of SsoFast EvaGreen Supermix (Bio-Rad). The thermocycling steps were 95°C for 30 s, 95°C for 5 s followed by 55°C for 5 s. To amplify the library, in PCR1, a 50 μl reaction mixture contained the following: 1 μl DNA template, 200 nM of each of P1 and P2, 200 μM dNTP mixture, 1× Taq buffer and 1.25 units of Taq DNA polymerase. The reaction was carried out for 15–20 cycles (94°C for 5 min; 94°C for 30 s, 55°C for 30 s and 72°C for 30 s). A gel/PCR DNA fragment extraction kit (IBI Scientific) was used to purify the PCR1 product. The purified product was used as the template for PCR2. One-tenth of the purified PCR1 product was further amplified for 12 cycles using P3 and P4 as the primers. A 200 μl PCR reaction mixture contains 4 μl diluted template, 250 nM each of P3 and P4, 200 μM dNTP mixture, 1× Taq buffer and 5 units of Taq DNA polymerase. The final PCR2 product was again purified by 10% dPAGE. The single-stranded FAM-labeled DNA was excised from the gel, ethanol precipitated and used as the library for the subsequent round of selection.

### Activity assay

Gel-based activity assays were performed with a final concentration of 0.7 μM of the FAM-labeled substrate strand and 1.1 μM of the enzyme. The DNAzyme complexes were prepared by annealing them in buffer C (50 mM MES, pH 6.0, 25 mM NaCl) and a final concentration of 10 μM Ln^3+^ was added to initiate the cleavage reaction. The products were separated on a denaturing polyacrylamide gel and analyzed using a Bio-Rad ChemiDoc MP imaging system. For pH-dependent activity assay, the 2-(N-morpholino)ethanesulfonic acid (MES) and 3-(N-morpholino)propanesulfonic acid (MOPS) buffers (50 mM with 25 mM NaCl) were used.

### Sensing

The sensing kinetics studies were carried out using a microplate reader (SpectraMax M3). The sensor complex was formed by annealing the FAM-labeled substrate and the quencher-labeled enzyme (1:1.5 ratio) in buffer C. In each well, 100 μl of the complex containing 50 nM FAM-labeled substrate was diluted in 10 mM HEPES (pH 7.5). One microliter of metal ion was added after 5 min of background reading and the signaling kinetics was monitored. Most assays were run in triplicates and error bars represent one times of standard deviation.

## RESULTS AND DISCUSSION

### *In vitro* selection

**Figure 1.**
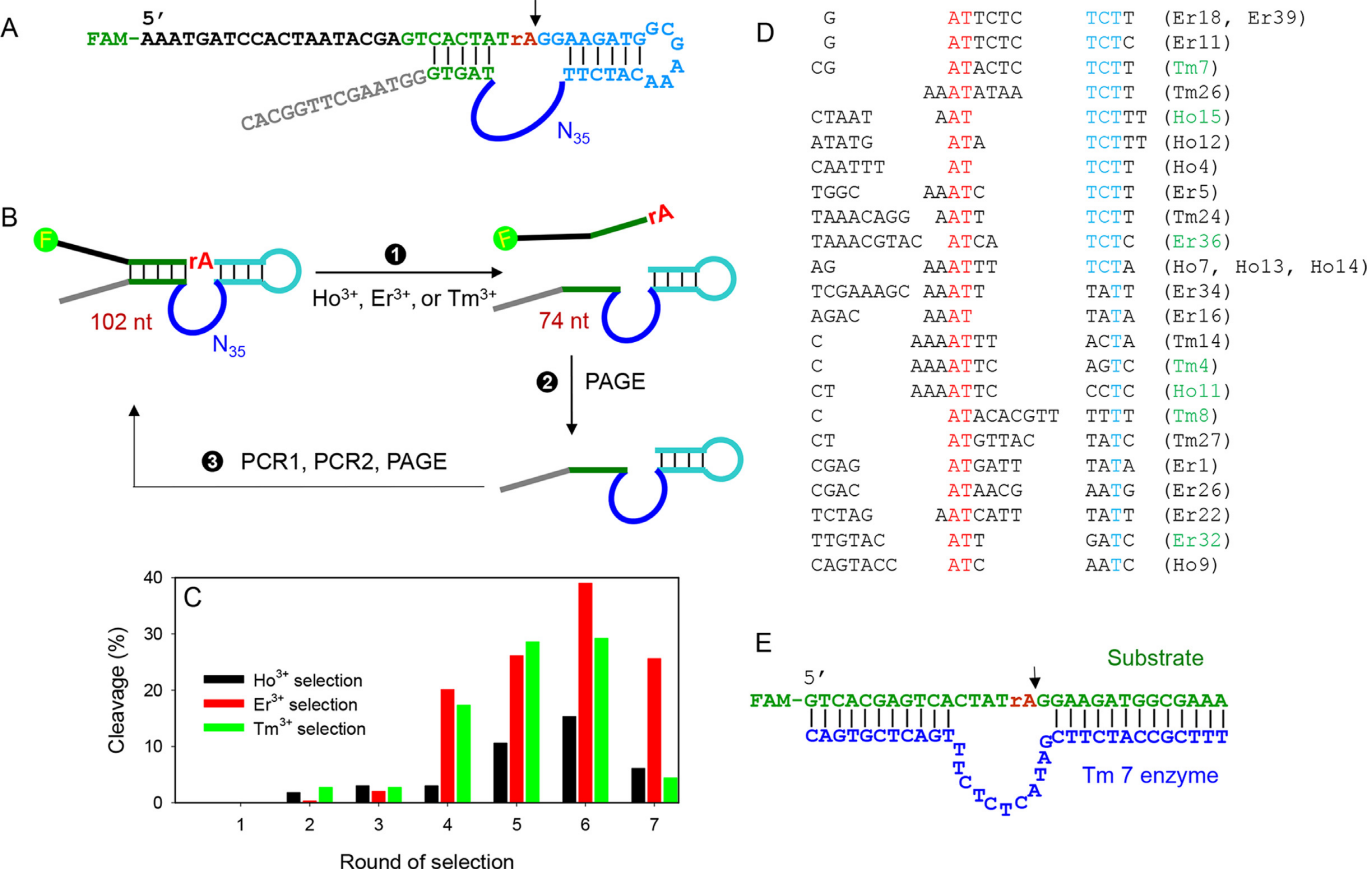
(**A**) The DNA library sequence. The cleavage site is indicated by the arrowhead. (**B**) A simplified scheme of the *in vitro* selection process. Three independent selections with the different Ln^3+^ were carried out. PAGE means gel electrophoresis and two PCR steps were used in step 3 to amplify and re-generate the library. (**C**) Selection progress and the round 6 library was sequenced. Ln^3+^ concentration was 50 μM for rounds 1–4 and 10 μM for rounds 5–7. Incubation time was maintained at 60 min. (**D**) Sequence alignment in the enzyme loop region. (**E**) The secondary structure of the trans-cleaving version of the Tm7 DNAzyme.

Free Ln^3+^ and some of their complexes can cleave RNA at high metal concentrations (1,17). We hope to use DNA as a scaffold to more efficiently use Ln^3+^ ions so that the same reaction takes place at much lower Ln^3+^ concentrations and at a designated position. *In vitro* DNAzyme selection provides a powerful tool to achieve this goal (18–23), and a number of metal-dependent DNAzymes have been isolated. In this work, selections were separately carried out using three different Ln^3+^ to increase sequence diversity. The design of our library is shown in Figure 1A. It contains a FAM (carboxyfluorescein) label at the 5′-end, so that the cleavage reaction can be quantified. The cleavage junction is the rAG (indicated by the arrowhead), where the rA (ribo-adenosine) is the only RNA linkage in this whole sequence. Since RNA is about one million fold less stable compared to DNA, cleavage is most likely to take place at this junction (24). The cleavage site is positioned in proximity to the randomized N35 loop (35 random nucleotides) and is flanked by two Watson–Crick base pairing regions. If any sequence in this library can cleave the RNA linkage in the presence of an added Ln^3+^, a shorter DNA is generated (from 102 nucleotides to 74, Figure 1B). This shortened piece is isolated after gel electrophoresis and amplified by two PCR steps to seed the next round of selection.

The selection progress is presented in Figure 1C. All the three selections experienced a steady improvement in cleavage yield until round 6. At this moment, 15–38% cleavage was achieved. Since the activity of the round 7 libraries dropped, the round 6 libraries were sequenced. We obtained a total of 60 sequences from the three selections and they were aligned altogether for a systematic comparison (Supplementary Table S2). The DNAzymes are named by the Ln^3+^ used in selection followed by the clone number for sequencing. Most of them can fold into a simple loop structure and the folding of Tm7 is shown in Supplementary Figure S1 as an example (predicted by Mfold) (25). Based on this, a trans-cleaving version was designed (Figure 1E).

Assuming such a simple loop structure flanked by two base paired regions, we aligned the sequences in the loop region in Figure 1D. However, it is difficult to identify a long-stretch of conserved nucleotides. This situation is quite different from our previously reported two selections (13,14). In those two cases, the conserved nucleotides were easily identified. In Figure 1D, the sequences are aligned based on an *AT* dinucleotide (marked in red) and a thymine in blue. About half of them end with *TCTT* (also marked in blue), and many contain a stretch of adenines in the middle prior to the conserved *AT* dinucleotide. The sequences in Figure 1D represent about 50% of the library. For the rest, 16 of them (∼27%) are the Lu12 type of DNAzyme we previously reported (14). It is not surprising that Lu12 was selected again since it is active with all these three lanthanide ions.

### Lanthanide selectivity

**Figure 2.**
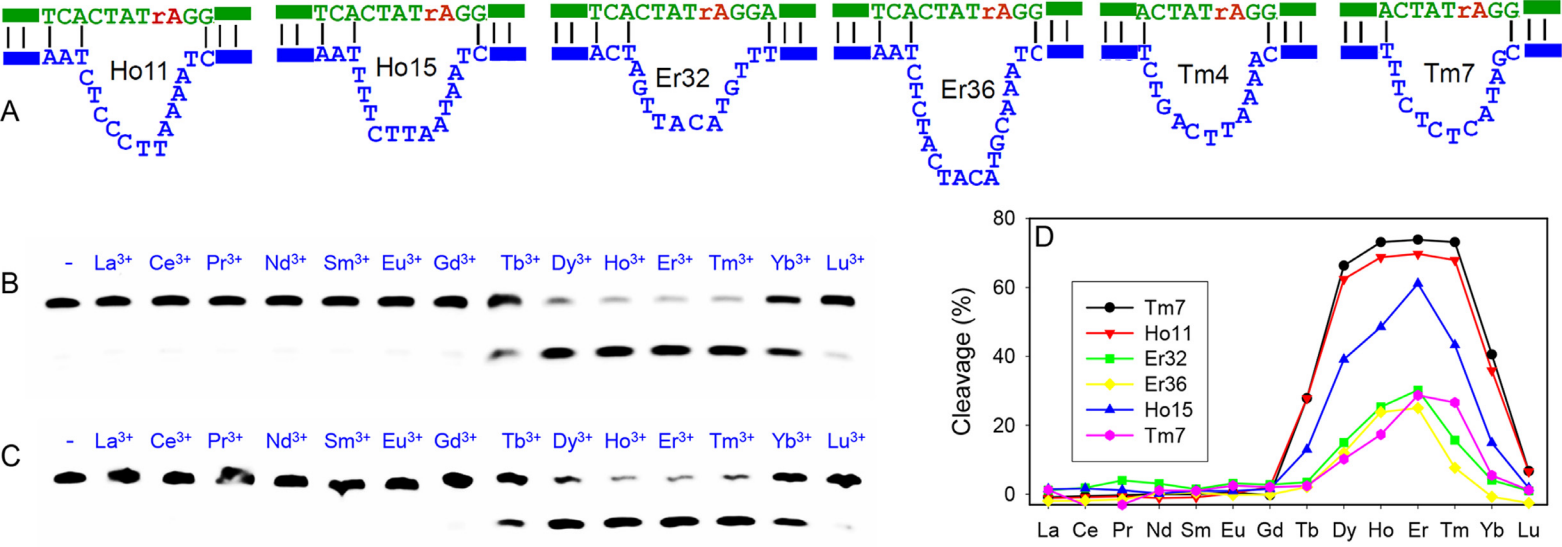
(**A**) Secondary structures of six DNAzymes in this study. Gel images showing cleavage activity of (**B**) Tm7 and (**C**) Ho11 with different Ln^3+^ ions (10 μM) after 1 h reaction. (**D**) Quantification of the cleavage results of the six DNAzymes by Ln^3+^.

Since the sequences of this new DNAzyme family are quite diverse, it is difficult to rationally predict the most optimal sequence. Therefore, we picked seven of them for further assay (those marked in green in Figure 1D). Each was designed in a trans-cleaving format, all sharing the same substrate as shown in Figure 1E. The DNAzyme secondary structures are shown in Figure 2A (substrate binding arms are denoted by the bars). All these DNAzymes contain a stretch of four unpaired nucleotides in the substrate strand at the 5′ side of the cleavage junction.

We next measured the enzyme activity as a function of Ln^3+^ atomic number after 1 h reaction. With a FAM label in the substrate, the cleavage reaction was conveniently followed using gel electrophoresis. Figure 2B and C show the trend for the Tm7 and Ho11 DNAzymes, respectively. Interestingly, hardly any cleavage was observed with the seven lighter ions (Pm^3+^ was not tested since it is radioactive) and only moderate cleavage was observed with Tb^3+^. Efficient cleavage occurred from Dy^3+^ to Tm^3+^, and then the cleaved product decreased rapidly with Yb^3+^ and Lu^3+^. This is a general trend for all the six tested DNAzymes and their activities are quantified in Figure 2D. Based on this study, we reason that these DNAzymes belong to the same family. From the sequence diversity in Figure 1D, they seem to be quite tolerant to mutations and insertions.

Tm7, Ho11 and Ho15 are the three fastest DNAzymes among the six. They all have a stretch of pyrimidines followed by a few purines in the loop (this is also true for Tm8, see Supplementary Figure S2). The other three DNAzymes are slower and their base contents in the loop region are more mixed up, especially on the pyrimidine side. It is also interesting to note that for the three most active sequences, only Tm7 has a single guanine in the loop, while Ho11 and Ho15 do not contain any guanines. Among the four nucleobases, guanine is the most efficient ligand for Ln^3+^ binding (7,26–28). The other two Ln^3+^-dependent DNAzymes are richer in guanine in their conserved sequences (13,14). This might be a reason for the completely different activity trends for these DNAzymes across the Ln^3+^ series.

The ionic radii of Ln^3+^ decrease gradually and steadily from 1.17 to 1.0 Å, while the p*K*_a_ values of their bound water decrease from 9.3 to 8.2 from La^3+^ to Lu^3+^. Given the abrupt change of the cleavage activity from Gd^3+^ to Tb^3+^, it is unlikely for either the size or p*K*_a_ to be the main factor. However, the ‘gadolinium break’ in Figure 2D may be attributed to the change of coordination number. As the lanthanide atomic number increases, the coordinated water decreases from 9 to 8 due to lanthanide contraction and steric effects (29). This transition takes place around Gd^3+^ for water ligand. It might be that the low coordination number allows better metal binding and catalysis in this DNAzyme.

Gadolinium break was reported previously with cleavage of a dinucleotide (5 mM lanthanide) (17), and with the GR5 DNAzyme (60 μM Ln^3+^) (10). In those two examples, however, Lu^3+^ was among the most active ions, but our Tm7 has almost no activity with Lu^3+^. Therefore, the mechanism is still different for Tm7. Since Tm7 has the highest efficiency among all the tested sequences and it has a small enzyme loop containing only 11 nucleotides, it was chosen for subsequent studies.

### Biochemical characterization of Tm7

**Figure 3.**
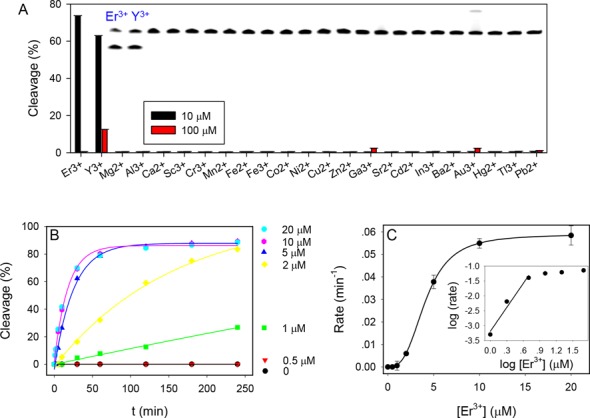
(**A**) Fraction of substrate cleavage by the Tm7 DNAzyme using 10 or 100 μM metal ions. Inset: gel image of cleavage in the presence of 10 μM metal ions. The lanes correspond to the metal ions in the *x*-axis. Only Au^3+^ produced streaking and Er^3+^ and Y^3+^ produced cleavage. (**B**) Kinetics of Tm7 cleavage at a few Er^3+^ concentrations. (**C**) Cleavage rate as a function of Er^3+^ concentration. Inset: the same data plotted using the log scale.

To further understand this new DNAzyme, we first tested metal selectivity for non-lanthanide ions. A gel image of Tm7 reacting with 10 μM other metal ions for 1 h is shown in the inset of Figure 3A. In addition to Er^3+^ (used as a representative Ln^3+^), only Y^3+^ produced cleavage, while all the other metals were inactive. The property of Y^3+^ is between Ho^3+^ and Er^3+^, which explains its activity. The same experiment was repeated with 100 μM metals (the red bars in Figure 3A), and still only Y^3+^ showed moderate cleavage. At 100 μM, even Er^3+^ inhibited the activity and we did not test even higher metal concentrations. It is important to note that Pb^2+^ was not active at all. Almost all the previously reported DNAzymes are active with Pb^2+^ (13,14,30,31). This is a rare example where Pb^2+^ failed to show activity. Such high selectivity makes Tm7 another useful probe for Ln^3+^, especially for the heavy ones.

Next, the cleavage kinetics was measured at various Er^3+^ concentrations (Figure 3B). With 0.7 μM DNAzyme, we did not observe any cleavage over 4 h in the presence of 0.5 μM Er^3+^. On the other hand, the rate increased by 10-fold from 1 to 2 μM Er^3+^. Since the rate does not scale linearly with the Er^3+^ concentration, it indicates that multiple metal binding is required for activity. Figure 3C plots the cleavage rate as a function of Er^3+^ concentration and a sigmoidal curve was obtained with a Hill coefficient of 3.0. In the inset, the double log plot is presented, where the initial slope is 2.7, suggesting that three Er^3+^ ions are involved in catalysis. The highest rate was ∼0.06 min^−1^ in the presence of 20 μM Er^3+^ at pH 6.0, which is similar to our selection condition. This is however slower than the rate of the Lu12 DNAzyme. The survival of this class of slower DNAzyme is attributed to that a long incubation time was used. They are also highly tolerant to mutations with good sequence diversity. At the moment of sequencing, after six rounds of selection we already have obtained 16 Lu12 DNAzymes out of 60 (27%). If we continue the selection with shorter time, the library is likely to be dominated by the Lu12 type of DNAzymes. Most of the sequences still have very low activity with Lu^3+^, and this might be the reason that we did not see any Tm7 sequences in our previous Lu^3+^-dependent selection (14). At higher pH, the rate of Tm7 increases significantly (see below) and can reach higher than 1.6 min^−1^.

To the best of our knowledge, this is the first time that metal cooperativity is reported for RNA-cleaving DNAzymes. All the previous reported enzymes employ only a single metal ion (10,13,14,31–34). Most small ribozymes use only one metal as well (35,36), although multiple metals are involved in large ribozymes performing more complex reactions such as RNA splicing (37,38). There is one example that Ln^3+^ (especially Nd^3+^) can accelerate the Pb^2+^-dependent activity of the leadzyme, where a two-metal mechanism was proposed. However, the best rate was only ∼0.01 min^−1^ when both metals were used (39). In addition, those two metals do not show much cooperativity.

Using multiple Ln^3+^ ions for RNA cleavage was reported with a high concentration (e.g. mM) of free ions (no DNAzymes) (1). It is proposed that one Ln^3+^ directly bonds with the leaving phosphate to decrease its negative charge density at the transition state. A bridging water (bridging two Ln^3+^ ions) may deprotonate and act as a general base to activate the 2′-OH of the ribose to make it a better nucleophile.

### The effect of pH

**Figure 4.**
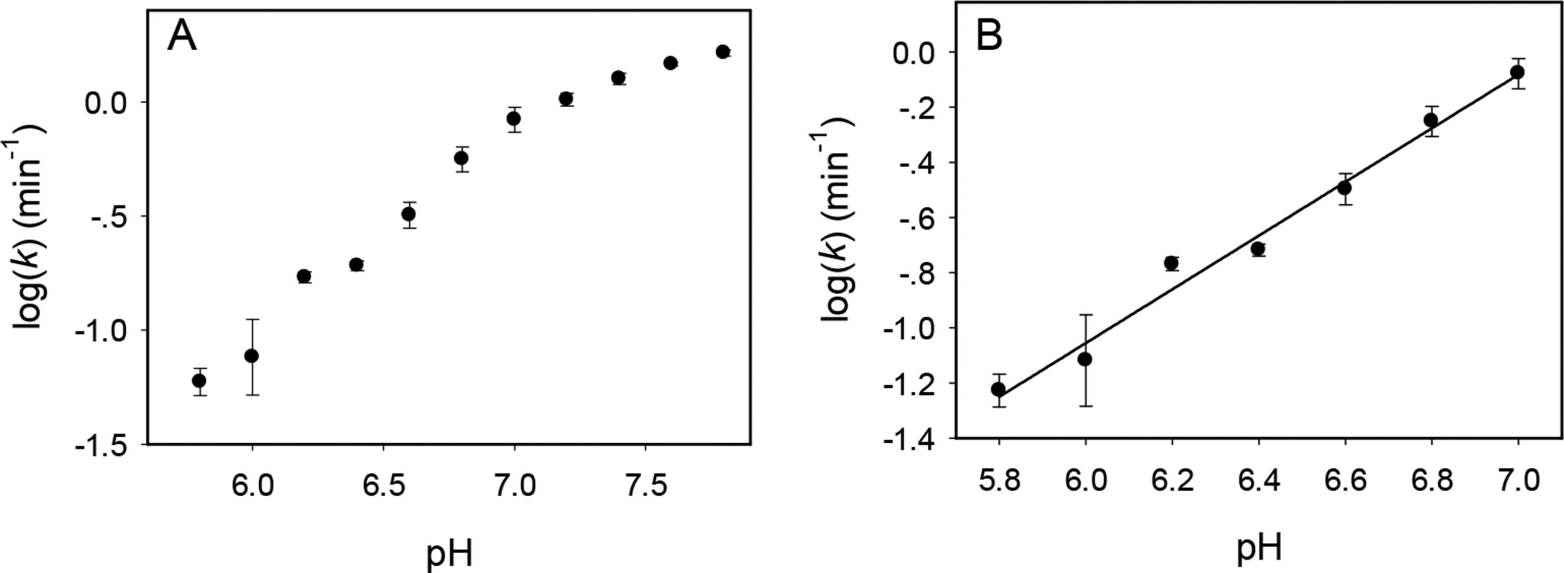
The pH-rate profile of the Tm7 DNAzyme over (**A**) a wide pH range and (**B**) the initial linear range. A slope of 0.97 is obtained in (B), indicating a single deprotonation step.

To gain further insights into Tm7, a pH-dependent study was performed, and the logarithm of cleavage rate was plotted (Figure 4A). While the selection was carried out at pH 6.0, the rate became progressively faster at higher pH (31,34,40). At pH higher than 7.0, the rate increase slowed down. By fitting the data in the pH range from 6.0 to 7.0, a slope of 0.97 was obtained (Figure 4B), suggesting only a single deprotonation step is involved in the rate-limiting step of the cleavage reaction. This is typical for RNA-cleaving DNAzymes and this deprotonation is often linked to the 2′-OH group on the ribose.

### Phosphorothioate modification

**Figure 5.**
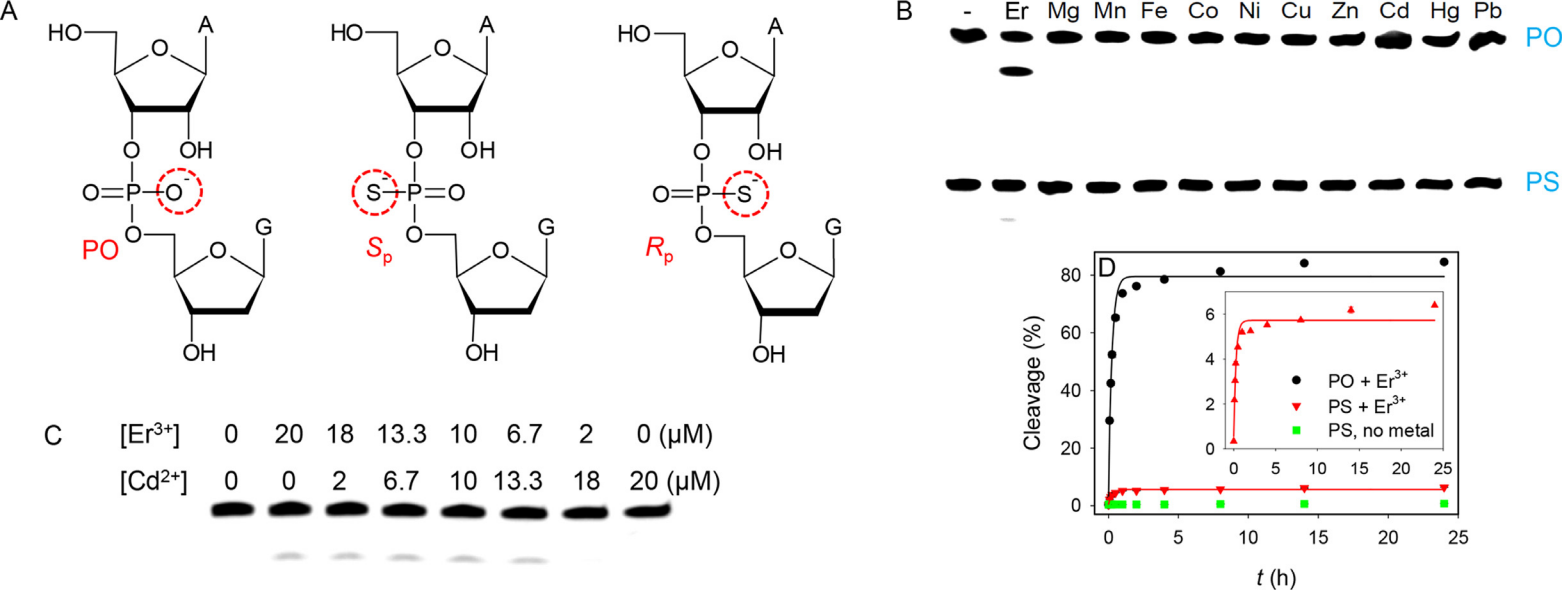
(**A**) Structures of the normal phosphate linkage (PO), and the two diastereomers of the PS modification. (**B**) Cleavage of the Tm7 DNAzyme with the PS-modified substrate (racemic mixture) in the presence of various divalent metal ions and Er^3+^. (**C**) Cleavage of Tm7 with the PS substrate using a mixture of Er^3+^ and Cd^2+^. (**D**) Kinetics over 24 h for Tm7 cleaving the PO (black dots), and PS (red triangles) substrate in the presence of 10 μM Er^3+^. The green squares are the Tm7/PS complex incubated without Er^3+^. Inset is the re-plot of the PS sample magnifying the initial kinetics.

Since lanthanide ions are hard Lewis acids that prefer oxygen-based ligands, we further probed metal binding by introducing a phosphorothioate (PS) modification at the cleavage junction, where one of the non-bridging oxygen atoms is replaced by a sulfur (Figure 5A) (41,42). Depending on the position of replacement, two diastereomers are possible. With the PS-modified substrate (a mixture of the two isomers), Tm7 barely showed cleavage with any metal, including thiophilic Cd^2+^ (Figure 5B), which is typically used to rescue the activity of PS-modified enzymes. Only Er^3+^ showed a trace amount of cleavage after 1 h. We performed a quantitative kinetic measurement (Figure 5D) and the initial kinetics is very similar to that of the PO substrate (within 10% difference) and no further cleavage occurred beyond ∼5%. Therefore, this small fraction of cleavage is attributed to that the PS substrate is only ∼95% pure and the remaining 5% are PO, explaining the observed Er^3+^-dependent initial cleavage kinetics.

All the previously studied ribozymes and DNAzymes (e.g. the hammerhead ribozyme (43), HDV ribozyme (44), 10–23 DNAzyme (45), and RNase P (46)) use the pro-*R*_p_ oxygen for metal binding (mostly Mg^2+^). Replacing this oxygen by sulfur usually completely inhibits the Mg^2+^-dependent activity (by over 100-fold), but this inhibition can be rescued by using thiophilic metals such as Mn^2+^ or Cd^2+^. The activity of the other PS isomer (*S*_p_) is only slightly decreased (e.g. ∼5-fold). This is because Mg^2+^ only coordinates to one of the non-bridging oxygen atoms (pro-*R*_p_), and thus the pro-*S*_p_ substitution does not have much influence on activity. For the Tm7 experiment, we used a racemic mixture of *R*_p_ and *S*_p_ (no separation performed). Since no activity was observed with either Er^3+^ or Cd^2+^, neither *R*_p_ nor *S*_p_ is active. In other words, the metal(s) must interact with both phosphate oxygen atoms through inner sphere coordination. This is different from all the previously reported nucleic acid enzymes, including another Ln^3+^-dependent DNAzyme, Ce13d, whose activity can be rescued by Cd^2+^ (47).

If both non-bridging oxygen atoms are important, we reason that a mixture of Er^3+^ and Cd^2+^ might rescue the activity if these two metals do not need to interact with each other. By fixing the total concentration at 20 μM, their ratio was varied (Figure 5C). Still only ∼5% cleavage was observed when the Er^3+^ concentration was more than 6.7 μM. This is again cleavage of the impure PO substrate. This implies that the metals must act synergistically instead of independently. If simply one metal binds to one oxygen, the use of this mixture should have boosted the activity with the PS substrate. The independent action of metal ions is best illustrated in the leadzyme, where a Ln^3+^ is proposed to bind to the phosphate oxygen and the Pb^2+^ is used to deprotonate the 2′-OH group (39). To the best of our knowledge, this is the first RNA-cleaving nucleic acid enzyme that is completely inactivated with the PS modification under all the metal conditions, which further confirms its new mechanism of catalysis.

### Cleavage mechanism

Before proposing mechanism, we performed mass spectrometry studies on the substrate cleavage product (Supplementary Figure S3). The 5′-fragment contains a cyclic phosphate, which is typical for most ribozymes and DNAzymes. We also tested the cleavage of a substrate with seven consecutive RNA bases (Supplementary Figure S4). However, Tm7 is inactive for this substrate. Most DNAzymes selected with a chimeric substrate are not active with full RNA. However, they are still called RNA-cleaving DNAzymes because of the same reaction mechanism.

**Figure 6.**
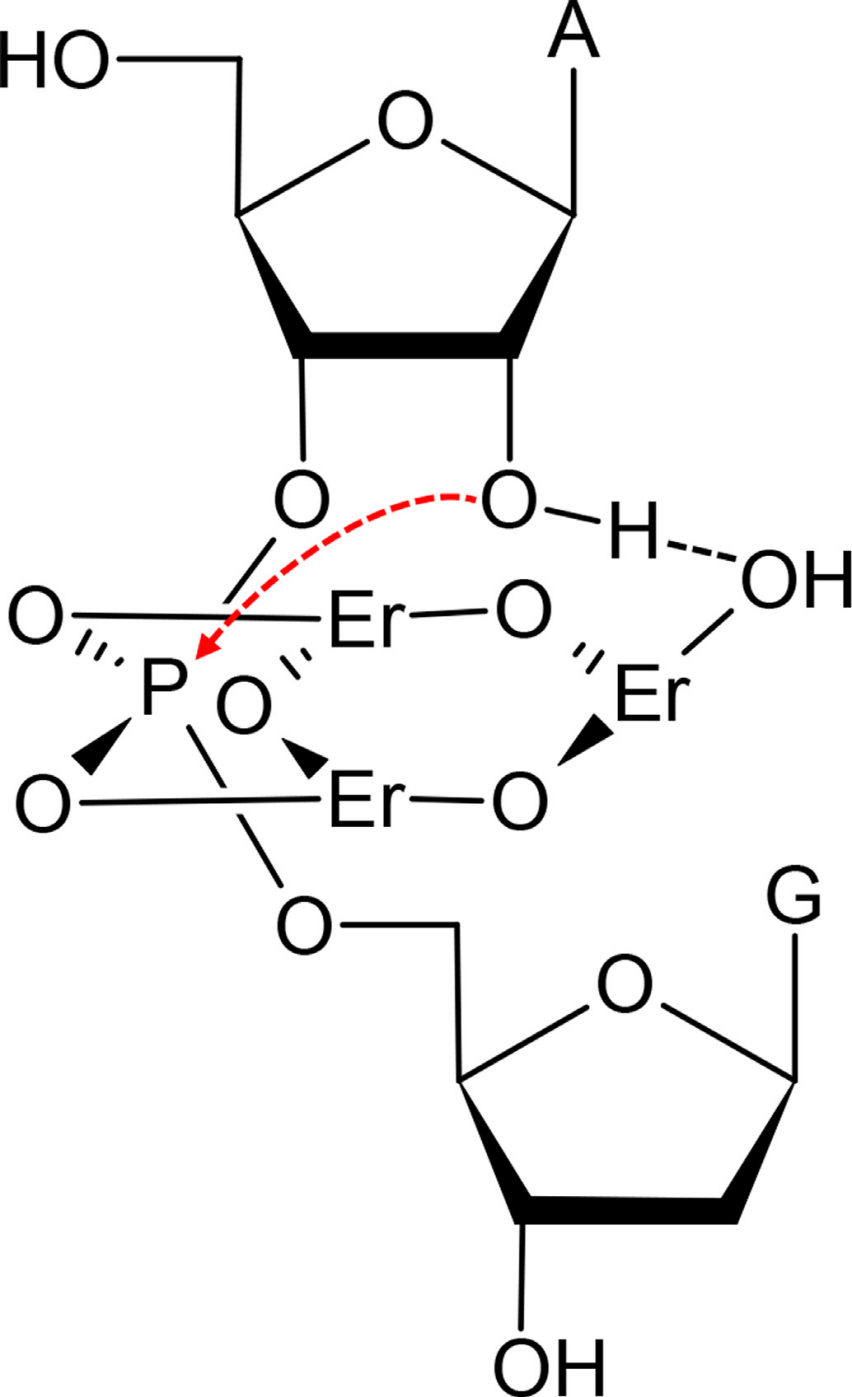
Proposed mechanism of the lanthanide-induced RNA cleavage for the Tm7 DNAzyme. The red arrow indicates nucleophilic attack of the phosphorus center. The bridging oxygen atoms linking the Er^3+^ ions are originated from deprotonated water.

Dinuclear Ln^3+^ complexes have been proposed to cleave RNA (1), which however cannot explain our results. Based on all the above studies, we propose a mechanism involving a trinuclear Ln^3+^ center (Figure 6). From the PS studies, both non-bridging oxygen atoms are in direct inner sphere coordination with Ln^3+^ (Er^3+^ used as an example here). These two can contribute to the electrostatic interactions to stabilize the transition state of the nucleophilic attack by the 2′-OH. Our pH studies indicate that only a single deprotonation reaction is involved, and this is likely to be related to the 2′-OH. Since the Ln^3+^ concentration-dependent studies indicate a total of three Ln^3+^ ions are involved, the third ion is thus proposed to interact with the 2′-OH. The three ions could be linked together with hydroxyl bridges. Since heavy Ln^3+^ ions have a lower p*K*_a_ value for the bound water, it is easier for them to form polynuclear hydrolyzed products, which could also explain the tendency of heavy Ln^3+^ being much more effective. The role of the DNAzyme loop is likely to stabilize this trinuclear complex.

### Sensing heavy lanthanide ions

Apart from proposing a novel reaction mechanism, this study has contributed a new probe for Ln^3+^ detection. Lanthanides are known as industrial vitamins since they are often doped at small quantities but are indispensable for modern technological applications. Rational design of small molecule ligands that selectively bind individual Ln^3+^ is quite difficult since these 15 elements have the same charge, similar sizes and comparable chemical properties (48). We are interested in developing DNA-based biosensors for Ln^3+^. DNA is a good candidate for Ln^3+^ ligand since the phosphate backbone provides high binding affinity through hard acid/base interactions and the nitrogen containing nucleobases may offer specificity to discriminate between different Ln^3+^ ions (49).

**Figure 7.**
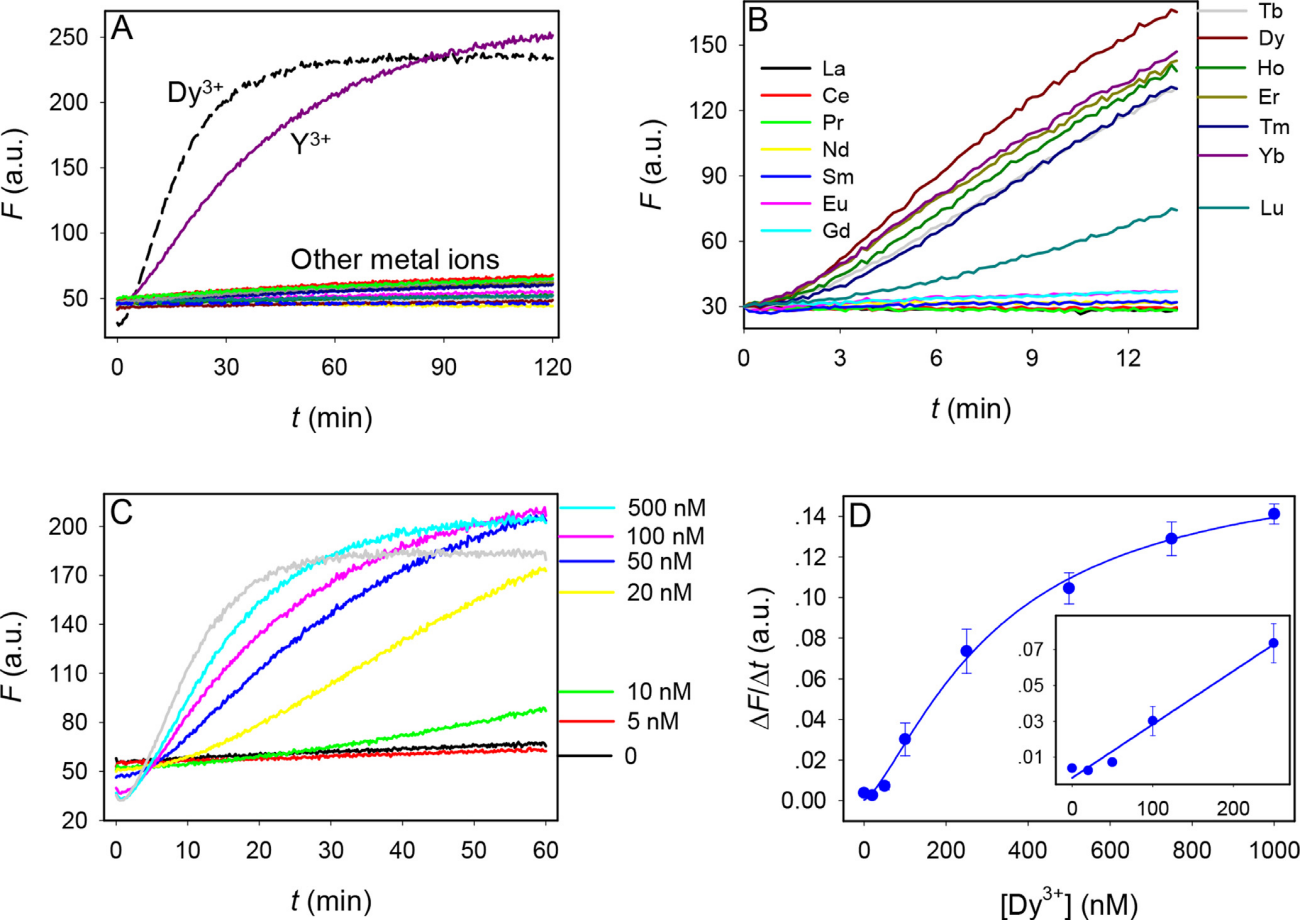
(**A**) Sensor response to 0.5 μM of divalent and trivalent metal ions. The list of the other metal ions tested can be found in Figure 2B. (**B**) Sensor response to 0.5 μM of various Ln^3+^. (**C**) Sensor signaling kinetics in the presence of various concentrations of Dy^3+^. DNAzyme sensor concentration = 50 nM. (**D**) Quantification of Dy^3+^ based on the initial rate of sensor fluorescence enhancement. Inset: the initial linear response at low Dy^3+^ concentrations.

To test the application of this DNAzyme for Ln^3+^ detection, we aim to engineer this DNAzyme into a biosensor. Although many sensor design methods are available (50,51), a simple catalytic beacon design was used (52). The 3′-end of the substrate labeled was labeled with a FAM and the 5′-end of the enzyme labeled with a quencher (Supplementary Figure S5). In the initial state, the beacon is dark. Fluorescence is unmasked upon cleavage and release of the cleaved fragment. Fluorescence enhancement was observed with Dy^3+^ and Y^3+^, but the non-rare earth metals failed to produce signal (Figure 7A). Among the lanthanide ions, only those seven heavy ones produce signal (Figure 7B). This is also consistent with the gel-based assays, indicating that the signal generation is indeed due to cleavage. Interestingly, the fastest signaling was observed with Dy^3+^. This might be attributed to the use of higher pH (7.5) in sensing as compared to pH 6.0 in the gel-based assay. We next performed a metal concentration dependency study using Dy^3+^ as the target metal ion (Figure 7C). Interestingly, barely any cleavage was observed with 5 nM Dy^3+^ and significant improvement was achieved with 10 and 20 nM Dy^3+^. This trend also confirms metal cooperativity. The Dy^3+^-concentration dependent rate of fluorescence increase is shown in Figure 7D and the detection limit is 14 nM Dy^3+^ based on the signal greater than three times of background variation.

## CONCLUSIONS

In summary, we carried out three independent *in vitro* selection experiments using Ho^3+^, Er^3+^ and Tm^3+^ as metal cofactors. Out of the 60 obtained sequences, half of them belong to a new family of DNAzyme, which are active to the seven heavy Ln^3+^ ions only. Importantly, this new DNAzyme shows metal cooperativity as indicated by metal concentration dependency and phosphorothioate replacement studies. Combined with the pH-rate profile, we propose a new mechanism involving a trinuclear Ln^3+^ complex. Two Ln^3+^ ions interact with the non-bridging oxygen atoms and the third one interacts with the 2′-OH. This is the first RNA-cleaving DNAzyme showing such a metal binding property. Finally, we engineered a catalytic DNA beacon that can detect the heavy Ln^3+^ such as Dy^3+^ down to 14 nM.

## SUPPLEMENTARY DATA

Supplementary Data are available at NAR Online.

SUPPLEMENTARY DATA
