# Supplementary material for: A new heavy lanthanide-dependent DNAzyme displaying strong metal cooperativity and unrescuable phosphorothioate effect
Source: Nucleic Acids Res. 2014 Dec 8;43(1):461–9. doi: 10.1093/nar/gku1296 (PMC4288186; doi:10.1093/nar/gku1296)
Supplement: SUPPLEMENTARY DATA [file supp_43_1_461__index.html]

A new heavy lanthanide-dependent DNAzyme displaying strong metal cooperativity and unrescuable phosphorothioate effect — SUPPLEMENTARY DATA 

# A new heavy lanthanide-dependent DNAzyme displaying strong metal cooperativity and unrescuable phosphorothioate effect

## SUPPLEMENTARY DATA

**Files in this Data Supplement:**

- SUPPLEMENTARY DATA
