# Supplementary material for: A new heavy lanthanide-dependent DNAzyme displaying strong metal cooperativity and unrescuable phosphorothioate effect
Source: Nucleic Acids Res. 2014 Dec 8;43(1):461–9. doi: 10.1093/nar/gku1296 (PMC4288186; doi:10.1093/nar/gku1296)
Supplement: SUPPLEMENTARY DATA [file supp_gku1296_nar-03117-f-2014-File009.pdf]

Supplementary Data

A New heavy lanthanide-dependent DNAzyme displaying strong metal cooperativity and unrescuable phosphorothioate effect

Po-Jung Jimmy Huang, Mahsa Vazin, Żaneta Matuszek and Juewen Liu*

Department of Chemistry, Waterloo Institute for Nanotechnology, University of Waterloo, Ontario, Canada, N2L 3G1

E-mail: liujw@uwaterloo.ca

Table S1. DNA oligonucleotides used for in vitro selection and assays.

**DNA Name**	**Sequence and modifications (from 5′ to 3′)**
Lib-FAM-N_{35}	pGGCGAAACATCTT$\mathbf{N_{35}}$TAGTGGGTAAGCTTGGCAC-**FAM**
Lib-rA	AATACGAGTCACTAT**rA**GGAAGAT
splint	AAGATGTTTCGCCATCTTCCTATAGTCCACCACCA
P1 primer	GTGCCAAGCTTACCG
P2 primer	CTGCAGAATTCTAATACGAGTCACTATAGGAAGATGGCGAAACA
P3 primer	**FAM**-AAATGATCCACTAATACGACTCACTAT**rA**GG
P4 primer	AACAACAACAAC-**iSp18**-GTGCCAAGCTTACCG
Tm4	TTTCGCCATCTTCAAAATTCAGTCTGACTCGTGAC
Tm7	TTTCGCCATCTTCGATACTCTCTTTGACTCGTGAC
Tm8	TTTCGCCATCTTCATACACGTTTTTTA ACTCGTGAC
Er32	TTTCGCCATCTTTTGTACATTGATCTGACTCGTGAC
Er36	TTTCGCCATCTTCTAAACGTACATCATCTCTAACTCGTGAC
Ho11	TTTCGCCATCTTCTAAAATTCCCTCTAACTCGTGAC
Ho15	TTTCGCCATCTTCTAATAATTCTTTTAACTCGTGAC
RNA-sub	GTCACGAGTCAC**rUrArUrArGrGrA**AGATGGCGAAA-**FAM**
Sub-FAM	GTCACGAGTCACTAT**rA**GGAAGATGGCGAAA-**FAM**
Tm7-Q	**Iowa Black ® FQ**-CGCCATCTTCGATACTCTCTTTGACTCGTGAC

Table S2. Sequence alignment for the three selection (aligned together).

Ho7	CTGCAG-AATTCTAATACGAGTCACTATAGGAAGATGGCGAAACATCT-----TAG--A-	51
Ho13	CTGCAG-AATTCTAATACGAGTCACTATAGGAAGATGGCGAAACATCT-----TAG--A-	51
Ho14	CTGCAG-AATTCTAATACGAGTCACTATAGGAAGATGGCGAAACATCT-----TAG--A-	51
Er5	CTGCAG-AATTCTAATACGAGTCACTATAGGAAGATGGCGAAACATCT-----TTGG-C-	52
Er1	CTGCAG-AATTCTAATACGAGTCACTATAGGAAGATGGCGAAACATCT-----TCGAGA-	53
Er16	CTGCAG-AATTCTAATACSAGTCACYMTAGGAAGATGGCGAAACATCT-----T--AGA-	51
Er18	CTGCAG-AATTCTAATACGAGTCACTATAGGAAGATGGCGAAACATCT-----TGA--T-	51
Er39	CTGCAG-AATTCTAATAC------CTATAGGAAGATGGCGAAACATCT-----TGA--T-	45
Er11	CTGCAG-AATTCTAATACGAGTCACTATAGGAAGATGGCGAAACATCT-----TGA--T-	51
Tm4	CTGCAG-AATTCTAATACGAGTCACTATAGGAAGATGGCGAAACATCT-----TCA--A-	51
Tm7	CTGCAG-AATTCTAATACGAGTCACTATAGGAAGATGGCGAAACATCT-----TCG--A-	51
Tm26	CTGCAG-AATTCTAATACGAGTCACTATAGGAAGATGGCGAAACATCT-----TAA--A-	51
Ho12	CTGCAG-AATTCTAATACGAGTCACTATAGGAAGATGGCGAAACATCT-----TATATG-	53
Tm27	CTGCAG-AATTCTAATACGAGTCACTATAGGAAGATGGCGAAACATCT-----TCTATG-	53
Ho9	CTGCAG-AATTCTAATACGAGTCACTATAGGAAGATGGCGAAACATCT-----TCAGTA-	53
Er34	CTGCAG-AATTCTAATACGAGTCACTATAGGAAGATGGCGAAACATCT-----TCGAAAG	54
Tm14	CTGCAG-AATTCTAATA-GAGTCACTAT-GGAAGATGGCGAAACATCT-----TCAAAA-	51
Ho2	CTGCAG-AATTCTAATACGAGTCACTATAGGAAGATGGCGAAACATCT-----TAGCTC-	53
Er22	CTGCAG-AATTCTAATACGAGTCACTATAGGAAGATGGCGAAACATCTTC---TAGAAT-	55
Ho6	CTGCAG-AATTCTAATACGAGTCACTATAGGAAGATGGCGAAACATCT-----TAATAA-	53
Er32	CTGCAG-AATTCTAATACGAGTCACTATAGGAAGATGGCGAAACATCT-----TTTGTA-	53
Er24	CTGCAG-AATTCTAATACGAGTCACTATAGGAAGATGGCGAAACATCT-----TTAAACA	54
Er30	CTGCAG-AATTCTAATACGAGTCACTATAGGAAGATGGCGAAACATCT-----TTAAA--	52
Ho4	CTGCAG-AATTCTAATACGAGTCACTATAGGAAGATGGCGAAACATCT-----TCAAT--	52
Ho11	CTGCAG-AATTCTAATACGAGTCACTATAGGAAGATGGCGAAACATCT-----TCTAA--	52
Er36	CTGCAG-AATTCTAATACGAGTCACTATAGGAAGATGGCGAAACATCT-----TTAAA--	52
Er4	CTGCAG-AATTCTAATACGAGTCACTATAGGAAGATGGCGAAACATCT-----TAGTA--	52
Tm16	CTGCAG-AATTCTAATACGAGTCACTATAGGAAGATGGCGAAACATCT-----TAAGA--	52
Tm32	CTGCAG-AATTCTAATACGAGTCACTATAGGAAGARGGCGAAACATCT-----TAAGA--	52
Er23	CTGCAG-AATTCTAATACGAGTCACTATAGGAAGATGGCGAAACATCC-----TCAAACT	54
Ho15	CTGCAG-AATTCTAATACGAGTCACTATAGGAAGATGGCGAAACATCT-----TCTAATA	54
Tm8	CTGCAG-AATTCTAATACGAGTCACTATAGGAAGATGGCGAAACATCT-----TCATACA	54
Ho1	CTGCAG-AATTCTAATACGAGTCACTATAGGAAGATGGCGAAACATCT-----TAGAGGT	54
Er6	CTGCAG-AATTCTAATACGAGTCACTATAGGAAGATGGCGAAACATCT-----TAGA---	51
Er13	CTGCAG-AATTCTAATACGAGTCACTATAGGAAGATGGCGAAACATCT-----TCGAAAA	54
Er31	CTGCAG-AATTCTAATACGAGTCACTATAGGAAGATGGCGAAACATCTT---ATAGGTAA	56
Er25	CTGCAG-AATTCTAATACGAGTCACTATAGGAAGATGGCGAAACATCT------TGGAGA	53
Er35	CTGCAG-AATTCTAATACGAGTCACTATAGGAAGATGGCGAAACATCTTAGGGGTAGTGC	59
Tm17	CTGCAG-AATTCTAATACGAGTCACTATAGGAAGATGGCGAAACATCTT--GAGAAGCAC	57
Er12	CTGCAG-AATTCTAATACGAGTCACTATAGGAAGATGGCGAAACATCTT----G--TATA	53
Er26	CTGCAG-AATT-TAATACGAGTCACTATAGGAAGATGGCGAAACATCTT----CGACATA	54
Er27	CTGCAG-AATTCTAATACGAGTCACTATAGGAAGATGGCGAAACATCTT----TTATAGA	55
Er33	CGCCCTTAATTCTAATACGAGTCACTATAGGAAGATGGCGAAACATCTT----TTATAGA	56
Tm1	CTGCAG-AATTCTAATACGAGTCACTATAGGAAGATGGCGAAACATCTTG---TACAAGA	56
Er9	CTGCAG-AATTCTAATACGAGTCACTATAGGAAGATGGCGAAACATCTT----TACAAGC	55
Er20	CTGCAG-AATTCTAATACGA-TCACTATAGGAAGATGGCGAAACATCTT----TACAAGC	54
Er10	CTGCAG-AATTCTAATACGAGTCACTATAGGAAGATGGCGAAACATCTT----TACAAGC	55
Tm3	CTGCAG-AATTCTAATACGAGTCACTATAGGAAGATGGCGAAACATCTT----TACAAGC	55
Tm22	CTGCAG-AATTCTAATACGAGTCACTATAGGAAGATGGCGAAACATCTT----TACAAGC	55
Er37	CTGCAG-AATTCTAATACGAGTCACTATAGGAAGATGGCGAAACATCTT----TACAAGC	55
Ho3	CTGCAG-AATTCTAATACGAGTCACTATAGGAAGATGGCGAAACATCTT----TACAAGC	55
Ho10	CTGCAG-AATTCTAATACGAGTCACTATAGGAAGATGGCGAAACATCTT----TACAAGC	55
Er2	CTGCAG-AATTCTAATACGAGTCACTATAGGAAGATGGCGAAACATCTT----TACAAGG	55
Er17	CTGCAG-AATTCTAATACGAGTCACTATAGGAAGATGGCGAAACATCTT----TACAAGG	55
Er28	CTGCAG-AATTCTAATACGAGTCACTATAGGAAGATGGCGAAACATCTT----TACAAGG	55
Ho8	CTGCAG-AATTCTAATACGAGTCACTATAGGAAGATGGCGAAACATCTT----TACAAGG	55
Er19	CTGCAG-AATTCTAATACGAGTCACTATAGGAAGATGGCGAAACATCTT----TACAAGG	55
Er38	CTGCAG-AATTCTAATACGAGTCACTATAGGAAGATGGCGAAACATCTT----TACAAGG	55
Ho5	CTGCAG-AATTCCAATACGAGTCACTATAGGAAGATGGCGAAACATCTT----TACGAGA	55
Er15	CTGCAG-AATTCTAATACGAGTCACTATAGGAAGATGGCGAAACATCTT----TGTATAC	55

```
Ho7           -AATTTTC---------TATAACGAGTTTGAT-CATGTACCGTTAGT--CGGTAAGCTTG 98
Ho13          -AATTTTC---------TATAACGAGTTTGAT-CATGTACCGTTAGT--CGGTAAGCTTG 98
Ho14          -AATTTTC---------TATAACGAGTTTGAT-CATGTACCGTTAGT--CGGTAAGCTTG 98
Er5           -AAATCTC---------TTTAACGAATTTAGT-AGTGTACCG-TAGT--CGGTAAGCTTG 98
Er1           -TGATTTA---------TATAACGAGTAAAGG-ACCGA--TTGTAGT--CGGTAAGCTTG 98
Er16          -CAAATTA---------TATAACGAGTATAGG-AGCGAGCACATAGT--CGGTAAGCTTG 98
Er18          -TCT-CTC---------TTTAACGAGTATCAGTGGCTRCAACGTAGT--CGGTAAGCTTG 98
Er39          -TCT-CTC---------TTTAACGAGTATCAGTGGCTCCAACGTAGT--CGGTAAGCTTG 92
Er11          -TCT-CTC---------TCTAACGAGTATCAGTGGCTCCACCGTAGT--CGGTAAGCTTG 98
Tm4           -AATTCAG---------TCTGACGCG-GTAGGTGGGTCCACCATAGT--CGGTAAGCTTG 98
Tm7           -TACTCTC---------TTTGACGTTCGTATAAACGAGACA-ATAGT--CGGTAAGCTTG 98
Tm26          -TATAATC---------TTTAACG--AGTAACCCAGACACATATAGT--CGGTAAGCTTG 97
Ho12          -ATATCT----------TTTAACGAGTATTAA--ACCATAAGATAGT--CGGTAAGCTTG 98
Tm27          -TTACTA----------TCTAACGAGAAGAGA--ACCATAA-CTAG---CGGTAAGCTTG 96
Ho9           -CCATCAA---------TCTAACGAGTGTTAG--AACGCAA-ATAGT--CGGTAAGCTTG 98
Er34          CAAATTTA---------TTTGACGAGAAT-----GCATACAAACAGT--CGGTAAGCTTG 98
Tm14          ---TTTAC---------TATAACGAGTGTTTA--ACGTGGAAATTAG--CGGTAAGCTTG 95
Ho2           ---AATCA--------GCACGCATGCGTGATT-ATAGTGAAG-TAGT--CGGTAAGCTTG 98
Er22          ---CATTT--------ATTTGACCACG-GATT-GCAGATTA--TAG---CGGTAAGCTTG 97
Ho6           ---CTTT---------ATCTAACGAAGTATTA-CTCAAAGAGGTAGT--CGGTAAGCTTG 98
Er32          ---CATTG--------ATCTGACTCACTCATG-CT-GTGGACCTAGT--CGGTAAGCTTG 98
Er24          GGAATTTC---------TTTAACTCGCTGTAG-GTG-GACT---AGT--CGGTAAGCTTG 98
Er30          --ACTTTA---------TTTGACTCGGAGGAA-GAG-AATTGGTAGT--CGGTAAGCTTG 97
Ho4           --TTATTC---------TTTGATGAGAAAAAG-GTGGAACTATTAGT--CGGTAAGCTTG 98
Ho11          -AATTCC---------CTCTAACTCGTACAAT-GAC-CCTTGTTAGT--CGGTAAGCTTG 98
Er36          -CGTACAT-----CATCTCTAACTCGTTGATA-GAA-C----TTAGT--CGGTAAGCTTG 98
Er4           -CCCAAAGGGAGGATGTTATAACTCGTATCT------C-----TAGT--CGGTAAGCTTG 98
Tm16          GCCGTCAG--------GTATGCCCTGTCAAAA-ACT-CGT--ATAGT--CGGTAAGCTTG 98
Tm32          GCCGTCAG--------GTATGCCCTGTCAAAA-ACT-CGT--ATAGT--CGGTAAGCTTG 98
Er23          GATATCAG--------AAGATCAACTCCTATA-ACT-CG----TAGT--CGGTAAGCTTG 98
Ho15          A-TTCTTT-----------TAACTCGATTATG-TAG-CGTATATAG---CGGTAAGCTTG 97
Tm8           CGTTTTTT-----------TAACTCGCT-ATG-TAT-GGAAGGTAG---CGGTAAGCTTG 97
Ho1           ACGTATAG---------AGACGAG-GTGACGCAATT----GAGTAGT--CGGTAAGCTTG 98
Er6           ---TATGG---------AAACGATTGTGAGGCTATATGACGAGGTAG--CGGTAAGCTTG 97
Er13          AGGTATCT---------GAGTTTATTTGATTCGTTG----AT-TAGT--CGGTAAGCTTG 98
Er31          TGGAAACT---------AA---TATTTGACTCGCTG----ATATAGT--CGGTAAGCTTG 98
Er25          GAGAAACT---------CAATTTATTTGACTCAATG----GGTTAGT--CGGTAAGCTTG 98
Er35          GTAGGACG---------AGATTTATTTGGCTCGTAG-------------CGGTAAGCTTG 97
Tm17          GAAGTGC----------AGGTTTATTTGACAAATAAT-------AGT--CGGTAAGCTTG 98
Er12          ACTTAAGT---------AGGTTATTTTAACGAGTATT----ATTAGT--CGGTAAGCTTG 98
Er26          ACGAATGT---------TACCCCCTTTAACGAGTA------ATTAGC--CGGTAAGCTTG 97
Er27          TCGATAGA---------GGATTTATTTGACAGGTAT------TTAGT--CGGTAAGCTTG 98
Er33          TCGATAGA---------GGATTTATTTGACAGGTAT------T-AGT--CGGTAAGCTTG 98
Tm1           ACGGGAGA---------GGGTATATTTAAATCGTAG------T--AG--CGGTAAGCTTG 97
Er9           ATCAGTAG-------------ATTGGAAATGCGGTT------ATAGTGACGGTAAGCTTG 96
Er20          ATCAGTAG-------------ATTGGAAATGCGGTT------ATAGTGACGGTAAGCTTG 95
Er10          ATCAGTAG-------------ATTGGAAATGCGGTT------ATAGTGACGGTAAGCTTG 96
Tm3           ATCAGTAG-------------ATTGGAAATGCGGTT------ATAGTGACGGTAAGCTTG 96
Tm22          ATCAGTAG-------------ATTGGAAATGCGGTT------ATAGTGACGGTAAGCTTG 96
Er37          ATCAGTAG-------------ATTGGAGATGCGGTT------ATAGTGACGGTAAGCTTG 96
Ho3           ACAAATAG-------------ATTGGAAATGCGGTT------ATAGTGACGGTAAGCTTG 96
Ho10          ACCGGACA---------AACTATGGGAGGAGCGGTT------ATAGT--CGGTAAGCTTG 98
Er2           AAGGGTCC---------A---CTATGCACAACGGTT------ATAGTGACGGTAAGCTTG 97
Er17          AAGGGTCC---------A---CTATGCACAACGGTT------ATAGTGACGGTAAGCTTG 97
Er28          AAGGGTCC---------A---CTATGCACAACGGTT------ACAGTGACGGTAAGCTTG 97
Ho8           AAGGGTCC---------A---CTATGCACAACGGTT------GTAGTGACGGTAAGCTTG 97
Er19          AAGGGTCC---------A---CTATGCACAACGGTT------ATAGTGGCGGTAAGCTTG 97
Er38          AAGTGTCC---------A---CTATGCACAACGGTT------ATAGTGACGGTAAGCTTG 97
Ho5           TAGAATGT---------TG--TACAGTCTAGCGGTT------ATAGT--CGGTAAGCTTG 96
Er15          CGGAAAGG---------TAAAATTAATTTAACGAGA------GTAGT--CGGTAAGCTTG 98
```

Note: the bold A in red is the cleavage site. The bases in blue are conserved for Lu12. The green T denotes for the starting of the sequence alignment in the randomized region.

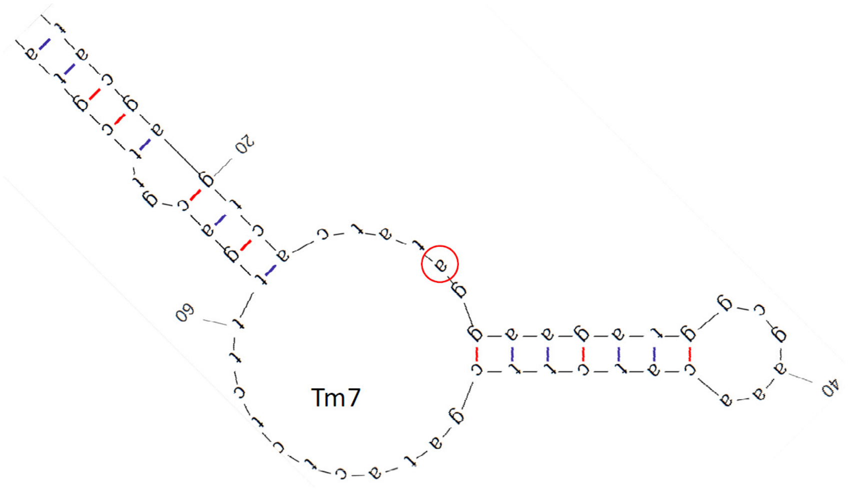

Figure S1. M-fold predicted Tm7 secondary structure in the cis-cleaving form. The numbering starts from the 5′-end of the sequence. The cleavage site rA is marked in the red circle. To engineer this into a trans-cleaving DNAzyme, the loop around nucleotide number 40 is removed and the base pairs are fixed as shown in Figure 1E of the main paper.

We tested a total of seven sequences in the family and six of them showed activity as presented in the paper. The last one, Tm8, is put here in SI due to limited space. The trans-cleaving Tm8 is presented in Figure S2A, which also has the pyrimidine stretch followed by a mixed purines and pyrimidines. However, it is also active active with the heavy lanthanides but not the lighter ones (Figure S2B).

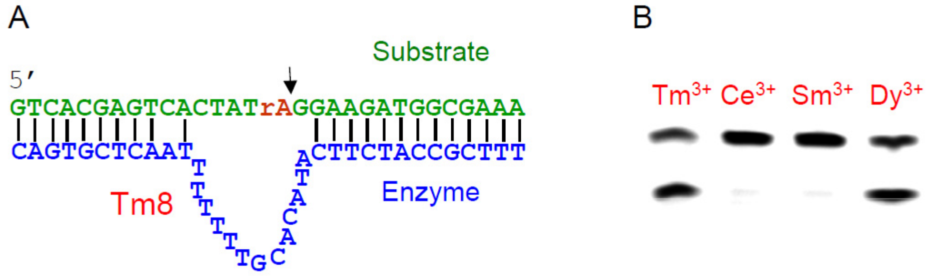

Figure S2. (A) The secondary structure of the trans-cleaving Tm8 DNAzyme. (B) Its activity in the presence of four lanthanides. Again, only the heavy lanthanides Tm^{3+} and Dy^{3+} produced activity.

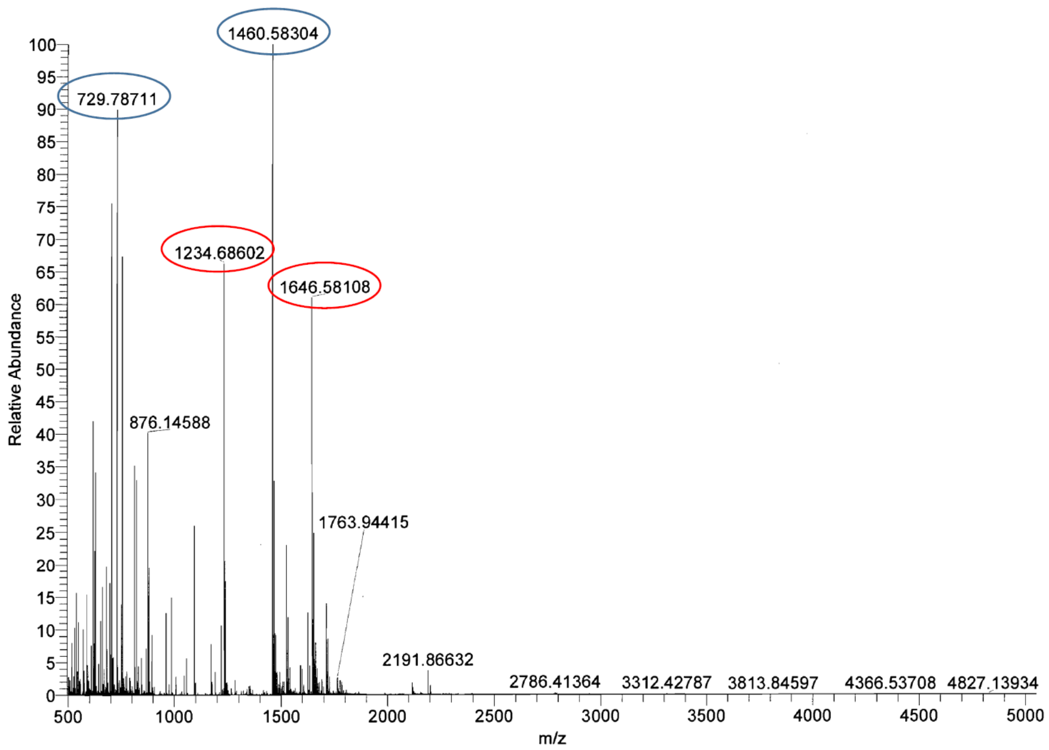

Figure S3. Mass spectrometry characterization of the Tm7 cleavage product. The peaks in red are the cyclic phosphate product from the 5′-fragment of the substrate (molecular weight = 4942.7, 3 and 4 charges for the two marked peaks), and the peaks in blue are from the 3′-fragment (molecular weight = 4384.7, 3 and 6 charges for the two marked peaks). These samples were prepared by reacting a non-labeled DNAzyme substrate with Tm7 or 17E DNAzymes at 1 μM substrate and 1.5 μM enzyme concentration in 50 mM MES buffer (pH 6) with 25 mM NaCl. Then the samples were desalted using a Sep-Pak column and dried. After rehydration in water to ~20 μM substrate concentration, the samples were analyzed using an ESI mass spectrometer.

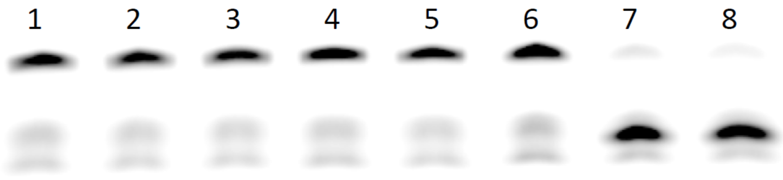

Figure S4. Tm7 and 17E cleavage of a RNA substrate (RNA-sub in Table S1 for sequence). Lane 1 is the substrate alone, and some degradation was observed. Lane 2 is the substrate with 10 μM Er^{3+}. Lane 3 is the Tm7 DNAzyme complex without metal. Lane 4 is the complex with 10 μM Er^{3+} for 30 min incubation. Lane 6 is the 17E DNAzyme complex without metal. Lane 7 and 8 are the 17E DNAzyme complex with 10 μM Pb^{2+} after 30 and 60 min incubation. The 17E DNAzyme is known to cleave full RNA, and it serves as a positive control. Cleavage was observed only with the 17E DNAzyme, but not Tm7.

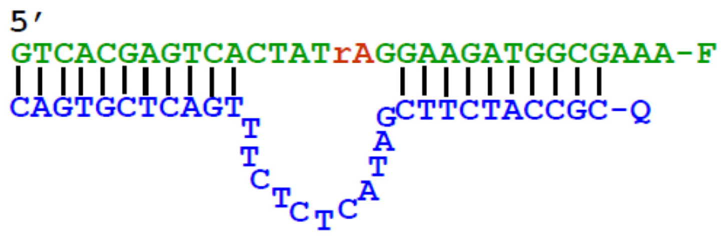

Figure S5. Catalytic beacon design using the Tm7 DNAzyme. F = FAM. Q = Iowa Black ® FQ dark quencher.
